# Supplementary material for: Overexpression of stress granule protein TZF1 enhances salt stress tolerance by targeting ACA11 mRNA for degradation in Arabidopsis
Source: Front Plant Sci. 2024 May 8;15:1375478. doi: 10.3389/fpls.2024.1375478 (PMC11122021; doi:10.3389/fpls.2024.1375478)
Supplement: Supplementary Data Sheet 1 — Transcriptome analysis. [file DataSheet_1.pdf]

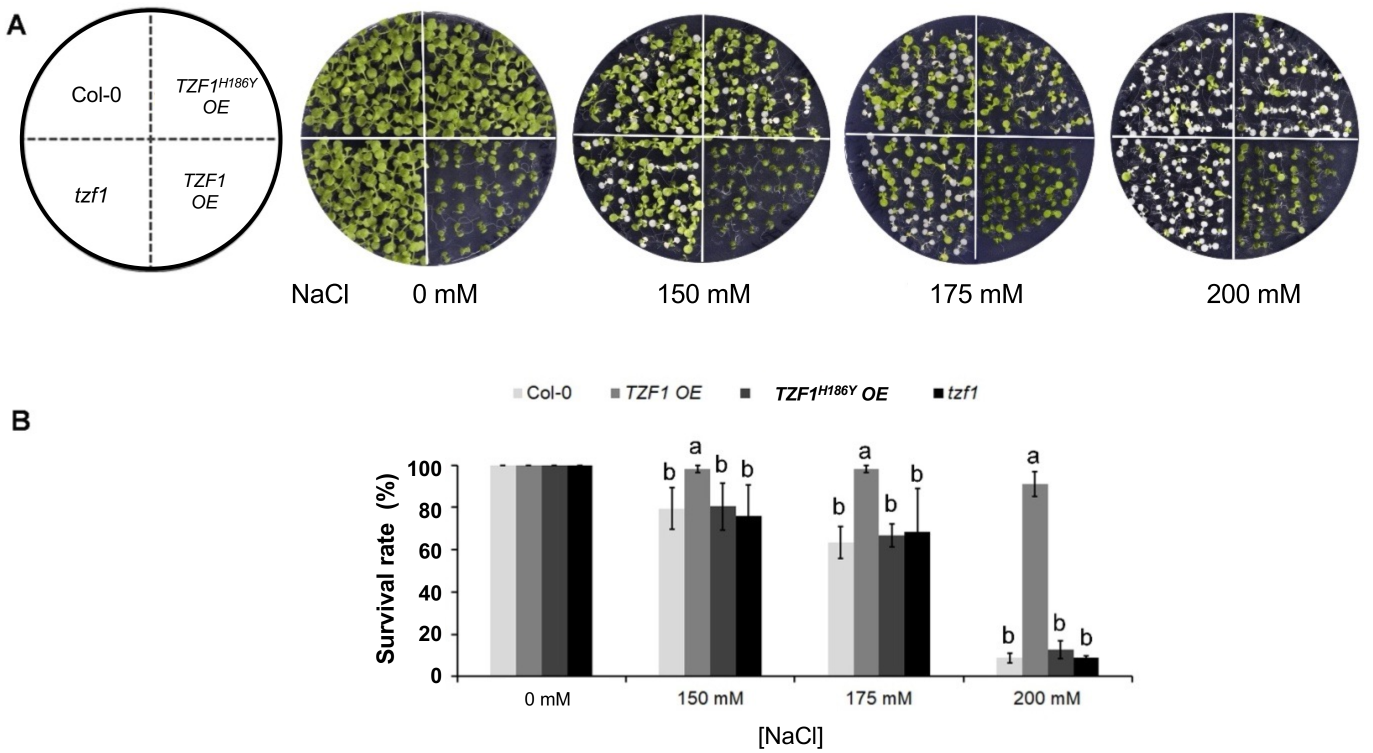

Supplementary Figure 1. The T-DNA knockout mutant *tzf1* showed salt stress-sensitive phenotypes.
(A) Phenotypes of the Col-0, *TZF1 OE*, *TZF1*^{H186Y} *OE*, and *tzf1* plants under normal and salt stress conditions. Seven-day-old seedlings grown under long-day condition (16/8 h light/dark cycles) were transferred to MS plates containing different NaCl concentrations for eight additional days. **(B)** Survival rates of seedlings shown in (A). Data represent the average of three replicates ± *SD*. Different letters (*a* and *b*) indicate significant differences at $P< 0.05$ by one-way ANOVA analysis performed using the SPSS software.

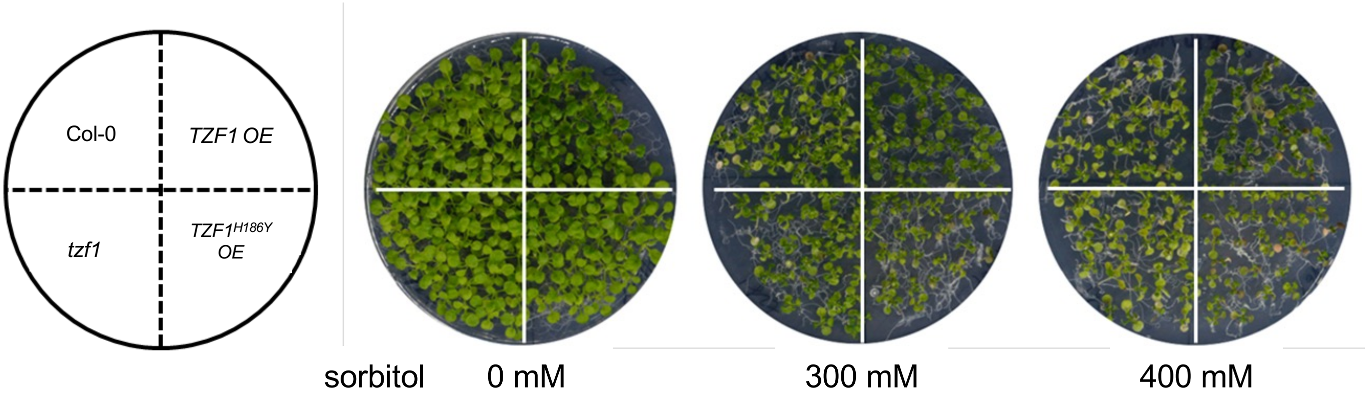

Supplementary Figure 2. TZF1 is not involved in the osmotic stress response induced by sorbitol. Phenotypes of the Col-0, *TZF1 OE*, *TZF1*^{H186Y} *OE*, and *tzf1* plants under osmotic stress induced by sorbitol. Seven-day-old seedlings grown under long-day condition (16/8 h light/dark cycles) were transferred to MS plates containing different concentrations of sorbitol and grown for eight additional days.

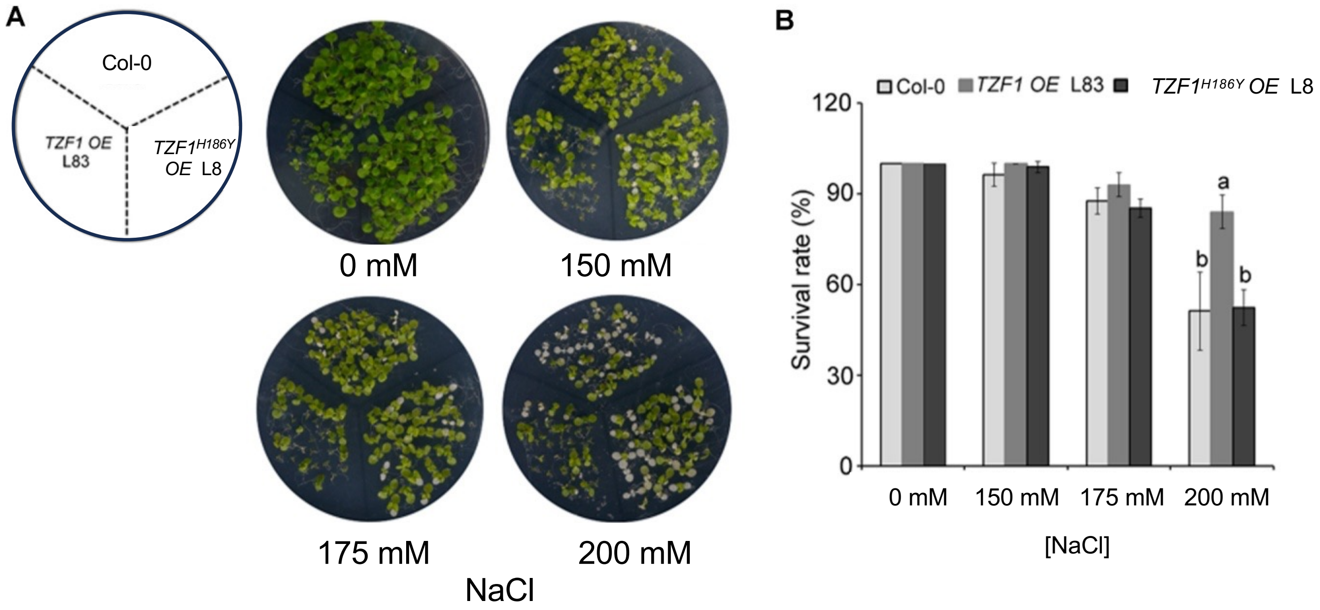

Supplementary Figure 3. Recapitulation of salt-stress phenotypes of EMS allele of *TZF1*^{H186Y} *OE* plants by ectopic expression of *TZF1*^{H186Y}*-GFP* in the wild-type background (*TZF1*^{H186Y} *OE L8*).

(A) Phenotypes of the Col-0, *TZF1 OE* (*L83*), and *TZF1*^{H186Y} *OE* (*L8*) plants under normal and salt stress conditions. Seven-day-old seedlings grown under long-day condition (16/8 h light/dark cycles) were transferred to MS plates containing different NaCl concentrations and grown for additional eight days. **(B)** Survival rates of seedlings shown in (A). Data represent the average of three replicates ± *SD*. Different letters (*a* and *b*) indicate significant differences at $P < 0.05$ by one-way ANOVA analysis performed using the SPSS software.

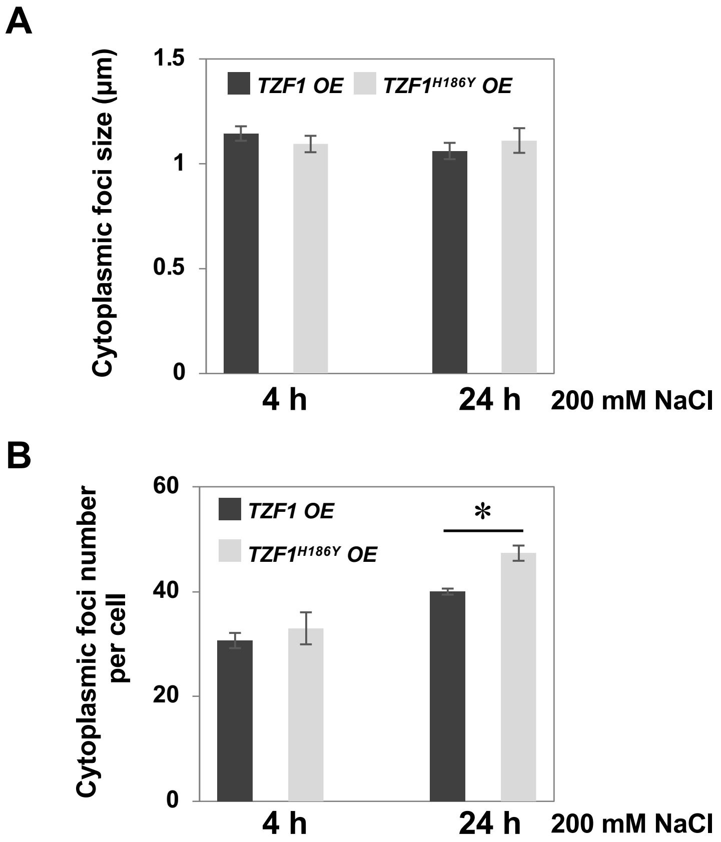

Supplementary Figure 4. Quantitative analysis of cytoplasmic foci size **(A)** and cytoplasmic foci number per cell **(B)** as shown in Figure 3. Bars represent means ± *SE* (n = 20). Asterisk indicates a significant difference between *TZF1 OE* and *TZF1*^{H186Y} *OE* (*, P < 0.05) by Student's *t* test.

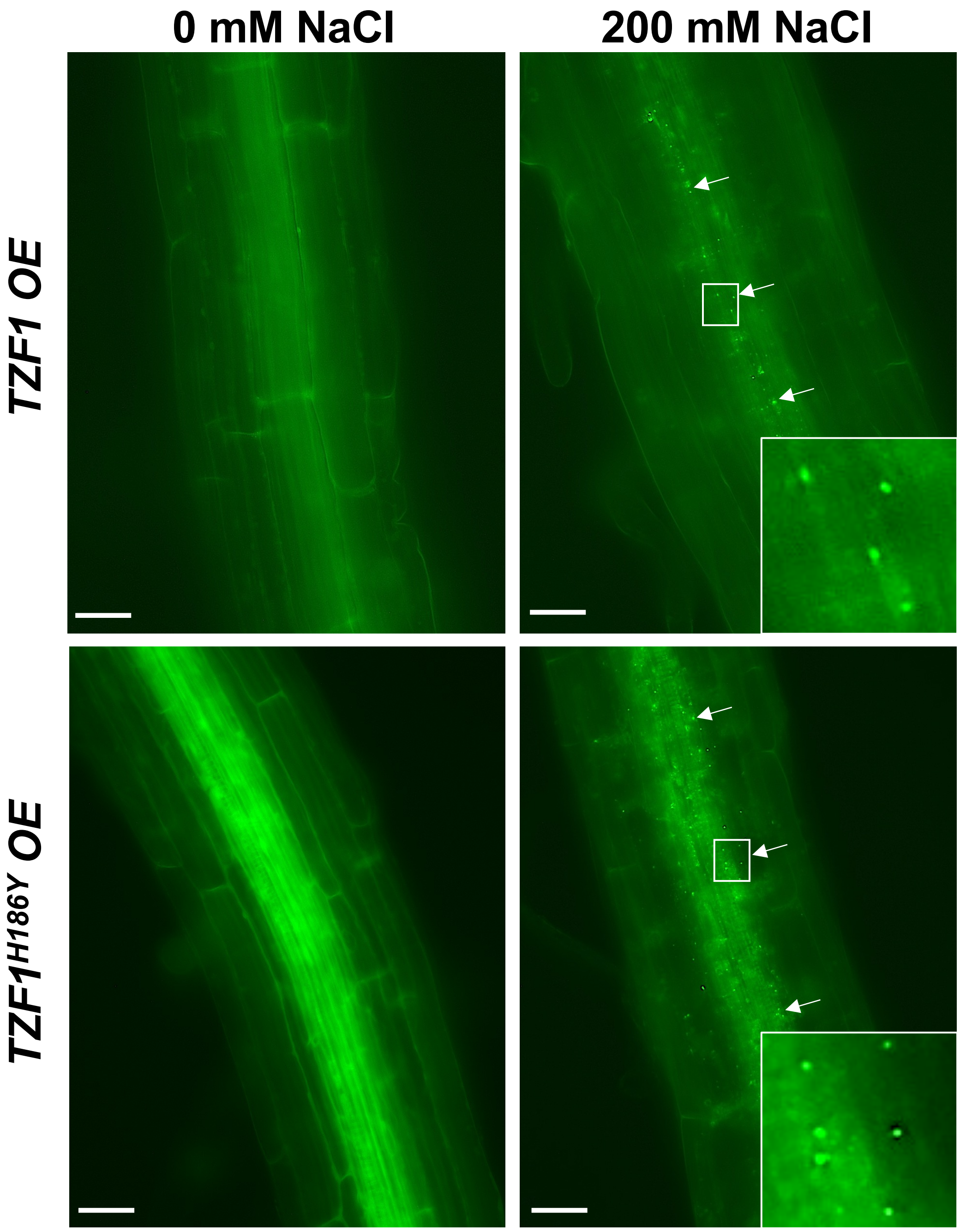

Supplementary Figure 5. TZF1 cytoplasmic foci induced by salt stress.

Fluorescent micrographs of root cells of seven-day-old *TZF1 OE* and *TZF1^{H186Y} OE* etiolated seedlings treated with 200 mM NaCl for 4 h. Consistent with the results shown in Figure 3, salt-induced cytoplasmic foci appeared to be more abundant in *TZF1^{H186Y} OE* plants.

A B

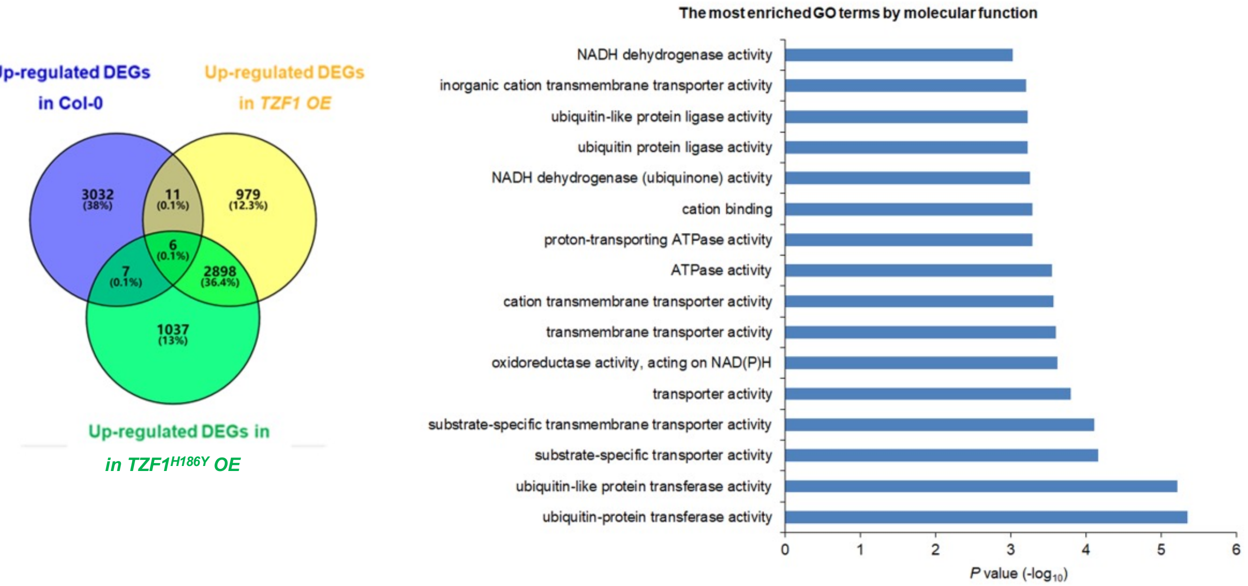

C

gene ID	gene name	description
AT3G12400	*VPS23A*	Ubiquitin-conjugating enzyme/RWD-like
AT1G80110	*PP2-B11*	F-box domain,Phloem protein 2-like
AT1G35720	*AnnAt1*	Annexin,Annexin D, plant,Annexin repeat
AT4G24400	*CIPK8*	NAF domain,Serine/threonine-protein kinase
AT5G17490	*RGL3*	Transcription factor GRAS,Transcriptional factor DELLA
AT3G62260	*PP2C*	PPM-type phosphatase, divalent cation binding
AT1G79610	*NHX6*	Na+/H+ exchanger,Cation/H+ exchanger
AT5G27150	*NHX1*	Na+/H+ exchanger,Cation/H+ exchanger
AT3G05030	*NHX2*	Na+/H+ exchanger,Cation/H+ exchanger

Supplementary Figure 6. RNA-seq analysis reveals up-regulated DEGs in responses to salt stress. **(A)** Venn diagram analysis of up-regulated DEGs in the Col-0, *TZF1 OE*, and *TZF1*^{H186Y} *OE* plants treated with NaCl. RNA-seq analysis was conducted using eleven-day-old seedlings grown on MS plates under 12 h light/dark cycles and treated with 150 mM NaCl for 4 h. **(B)** GO analysis of up-regulated DEGs specifically found in NaCl-treated *TZF1 OE* plants. *P* values were generated by Fisher's exact test. **(C)** A subset of up-regulated DEGs encoding positive regulators of salt stress tolerance found in NaCl-treated *TZF1 OE* plants.

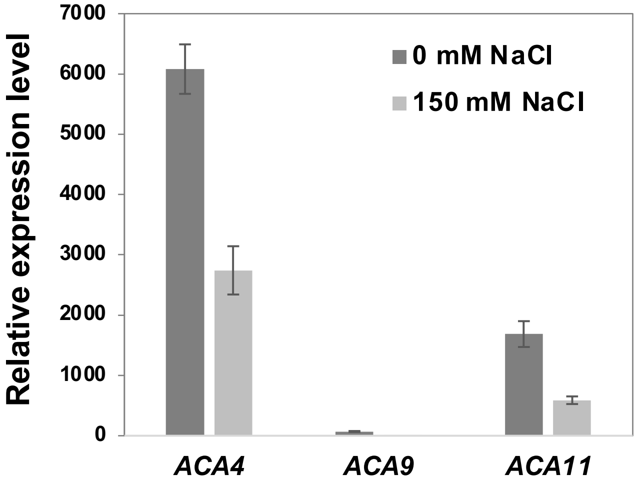

Supplementary Figure 7. *ACA4*, *ACA9*, and *ACA11* are downregulated in *TZF1 OE* plants under salt stress.

Normalized relative expression of *ACA4*, *ACA9*, and *ACA11* was determined from three biological RNA-seq analysis replicates.

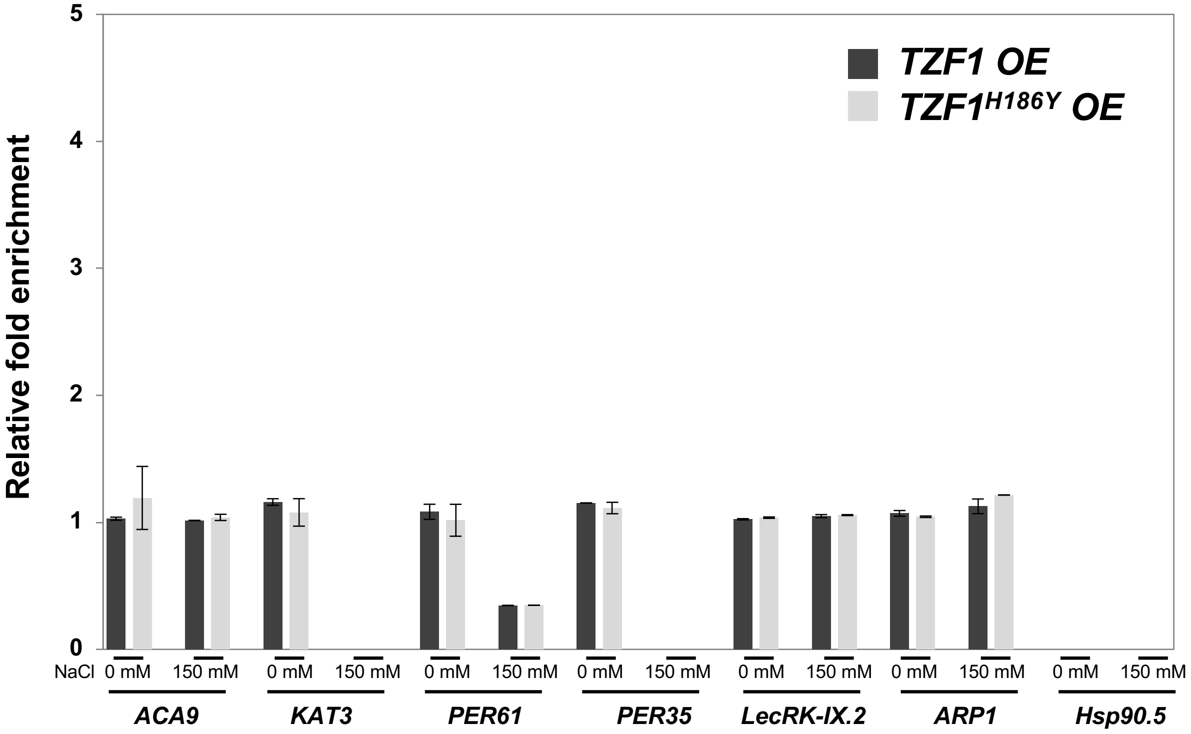

Supplementary Figure 8. RIP-qPCR analysis of potential TZF1 mRNA targets. Columns represent means± *SD*. No statistical differences were found between *TZF1 OE* and *TZF1*^{H186Y} *OE* in all comparisons as assessed by Student's *t* test ($P < 0.05$).

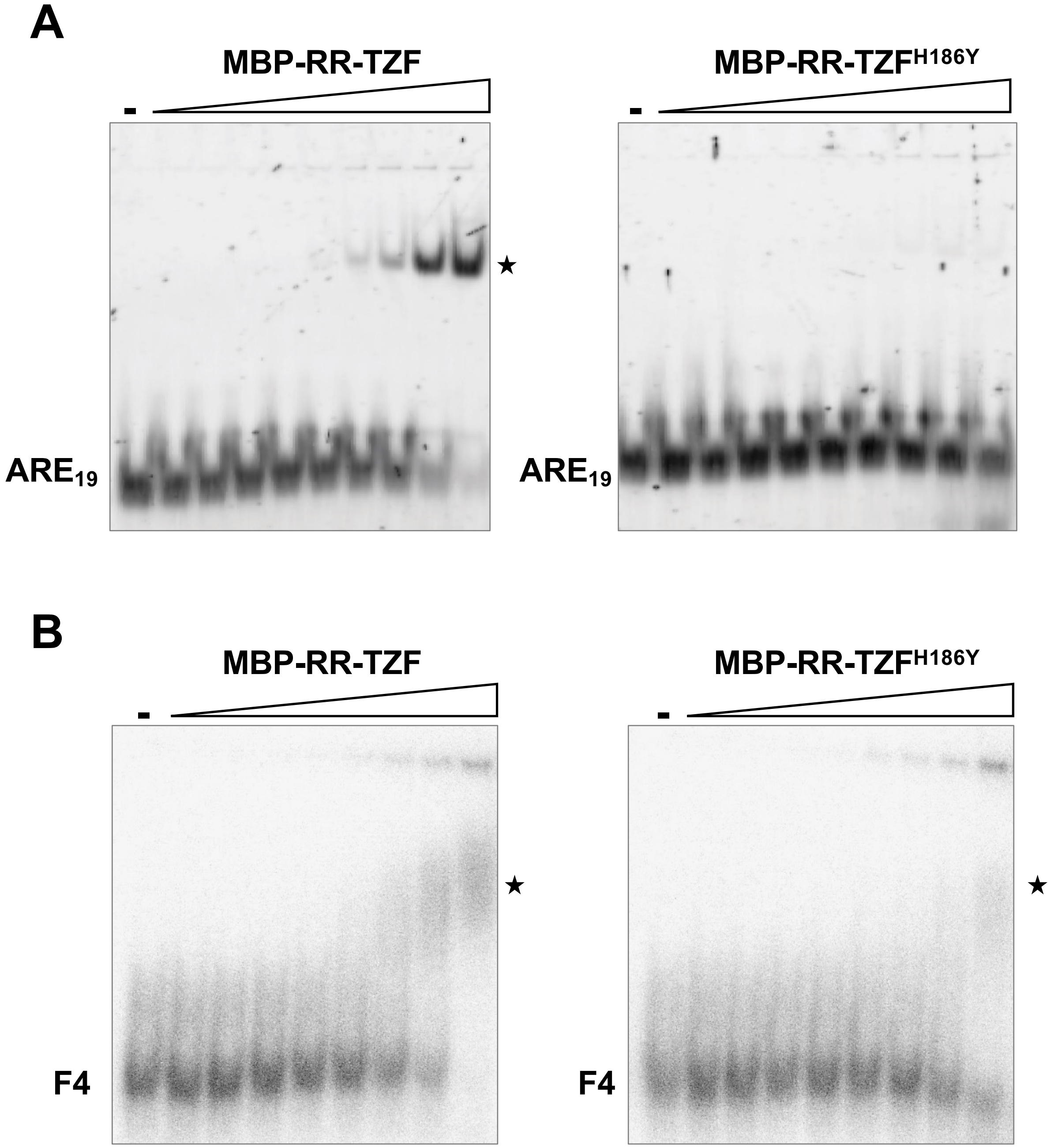

Supplementary Figure 9. The RR-TZF fragment of TZF1 protein binds to ARE_{19} and ACA11 F4 RNA probes in EMSA.

(A) The recombinant MBP-RR-TZF protein binds to ARE_{19} in EMSA. MBP-RR-TZF or MBP-RR-TZF^{H186Y} mutant (78 nM to 20 μM final) was incubated with 50 nM 6-FAM-ARE_{19} at 23˚C for 10 min, and complex formation was subsequently analyzed by native 8% (w/v) polyacrylamide gel electrophoresis. The RNA-protein complexes are indicated by an asterisk. **(B)** The recombinant MBP-RR-TZF protein binds to ACA11 F4 in EMSA. MBP-RR-TZF or MBP-RR-TZF^{H186Y} mutant (78 nM to 20 μM final) was incubated with 0.5 nM 5′-[^{32}P]-F4 RNA at 23˚C for 10 min, and complex formation was subsequently analyzed by native 6% (w/v) polyacrylamide gel electrophoresis. The intensity of the shifted band was higher for the MBP-RR-TZF protein than for the MBP-RR-TZF^{H186Y}, indicating tighter binding of F4 by the RR-TZF protein. The RNA-protein complexes are indicated by asterisks.

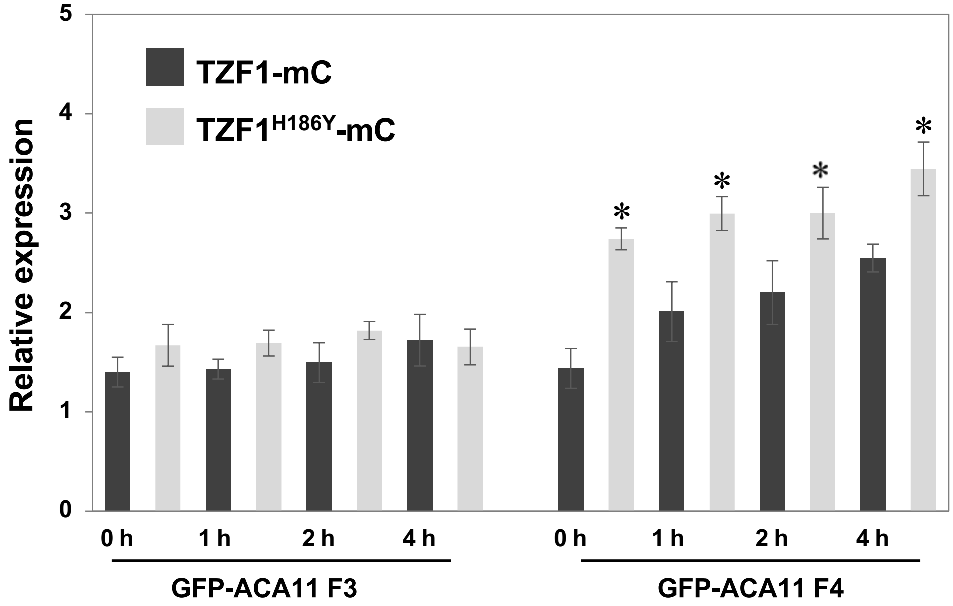

Supplementary Figure 10. Quantitative analysis of protein bands as shown in Figure 8E. Each bar value represents GFP-ACA11 F3 or F4 normalized with corresponding TZF1-mC or TZF1^{H186Y}-mC, respectively. Data represent the average of three replicates ± *SD*. Asterisk indicates a significant difference between TZF1-mC and TZF1^{H186Y}-mC (*, $P < 0.05$) by Student's *t* test.

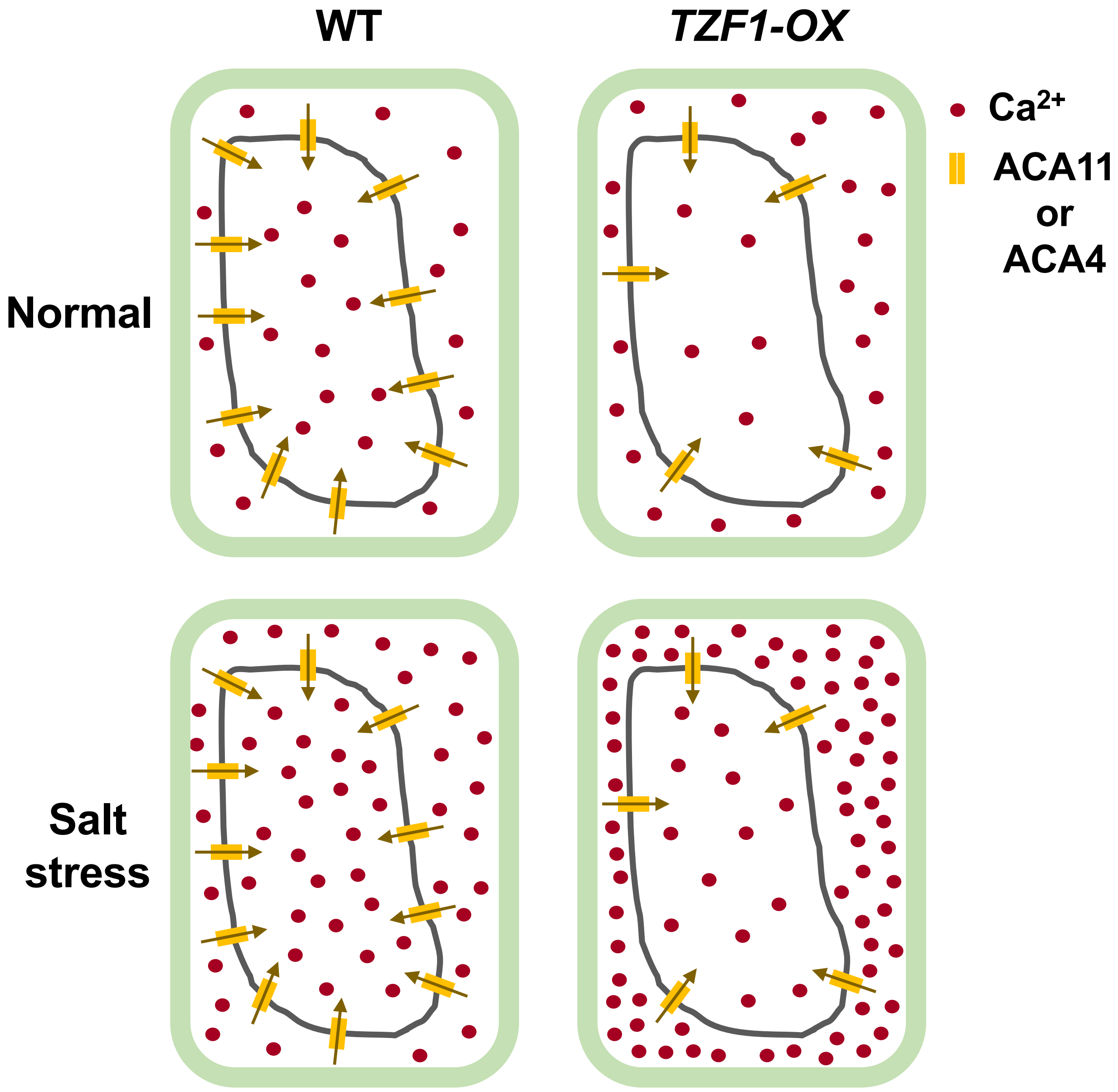

Supplementary Figure 11. Hypothetical model of increased Ca^{2+} distribution in the cytoplasm under salt stress in *TZF1 OE* plants.

Salt stress induces cytoplasmic Ca^{2+} accumulation. ACA11 and ACA4 are two major tonoplast-localized calcium pumps that expel Ca^{2+} from cytoplasm into the large central vacuole during salt stress. In *TZF1 OE* plants, decreased expression of *ACA11* hinders the calcium transport into the vacuole hence maintaining high Ca^{2+} concentration in the cytoplasm, supporting an enhanced salt stress tolerance response.

Supplementary Table S1. DNA oligonucleotides used in this study.

**Primers used for RIP-qPCR**	
ACA11-RIP-F1 F	5'-CCTTCTGGTCCATCTGACTCTG-3'
ACA11-RIP-F1 R	5'-ATCAGATCATTAGCCCGGAG-3'
ACA11-RIP-F2 F	5'-AACTCTTTCGTCTTTTGCCAG-3'
ACA11-RIP-F2 R	5'-AACAAACACCCAACTCTTGAAC-3'
ACA11-RIP-F3 F	5'-GAATGAGAAGAAGAGATGCCAG-3'
ACA11-RIP-F3 R	5'-ATCAGTGAGTTTATATTCAGGGC-3'
**Primers used for RT-PCR**	
ACTIN1 F	5'-CATCAGGAAGGACTTGTACGG-3'
ACTIN1 R	5'-GATGGACCTGACTCGTCATAC-3'
GFP F	5'-ATGGTGAGCAAGGGCG-3'
GFP R	5'-CTTGTACAGCTCGTCCA-3'
**Primers used for molecular cloning**	
ACA11 F3 for TOPO F	5'-CACCGAGAATGAGAAGAAGAGATGCCAG-3'
ACA11 F3 for TOPO R	5'-TCAATCAGTGAGTTTATATTCAGGGC-3'
ACA11 F4 for TOPO F	5'-CACCCCTTCTGGTCCATCTGACTCTG-3'
ACA11 F4 for TOPO R	5'-TCACAAGTGGAAACTCAGTTGAC-3'
TZF1 for TOPO F	5'-CACCATGATGATCGGCGAAAATAA-3'
TZF1 for TOPO R	5'-ACCGAGTGAGTTCTCTCTACTG-3'
**Primers used for RNA probes**	
ACA11 F1/F4 F	5'-ATGCCTAATACGACTCACTATAGG CCTTCTGGTCCATCTGACTCTG-3'
ACA11 F1 R	5'-GATCAGATCATTAGCCCGGAG-3'
ACA11 F4 R	5'-AAGTGGAAACTCAGTTGACA-3'
